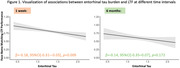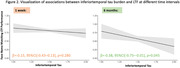# Characterizing accelerated long‐term forgetting in preclinical Alzheimer’s disease using the Boston Remote Assessment for NeuroCognitive Health (BRANCH)

**DOI:** 10.1002/alz.090570

**Published:** 2025-01-03

**Authors:** Cassidy Molinare, Daniel Soberanes, Mark A. Dubbelman, Stephanie Hsieh, Keith A Johnson, Reisa A Sperling, Dorene M Rentz, Gad A Marshall, Rebecca E Amariglio, Kathryn V Papp, Roos J Jutten

**Affiliations:** ^1^ Brigham and Women’s Hospital, Harvard Medical School, Boston, MA USA; ^2^ Massachusetts General Hospital, Harvard Medical School, Boston, MA USA

## Abstract

**Background:**

Accelerated long‐term forgetting (LTF) is characterized by unimpaired retention of information after short‐term delays (e.g., 20‐30 minutes) with increased forgetting at longer intervals (e.g., weeks to months). Previous studies have suggested that assessing LTF may provide a useful marker of preclinical Alzheimer’s disease (AD). However, assessing LTF over longer intervals is challenging using standardized in‐clinic paper‐pencil cognitive tests. Here, we leverage remote, digital cognitive testing to investigate LTF at different timepoints and its association with AD biomarkers in cognitively unimpaired (CU) older adults.

**Method:**

N = 61 CU older adults (age = 76.5±8.5, 67.2% female, 18% Aβ+) with amyloid (PiB) and tau (FTP) PET completed the Boston Remote Assessment for NeuroCognitive Health (BRANCH) at‐home on a personal device for seven consecutive days, including a Face‐Name Matching Task with identical stimuli each day. Learning across seven days was quantified using a previously validated multi‐day learning curve (MDLC) metric. Participants were asked to recall previously learned face‐name pairs after 1‐week (Median = 8(IQR = 7‐35) days) and after 6 months (Median = 6.67(IQR = 6.35,7.85) months). LTF was computed by dividing the percentage of correctly recalled face‐name pairs by a participant’s maximum performance during the 7‐day learning phase. We used linear regression models to examine the associations between LTF and initial MDLCs, global amyloid burden, entorhinal cortex (EC) and inferior‐temporal (IT) tau deposition, correcting for age, sex, and education when needed (covariates with a p‐value > 0.1 were excluded).

**Result:**

Better initial MDLCs were associated with less accelerated LTF after 1 week (β = 0.55,95%CI[0.00‐1.09],*p* = 0.048), but not after 6 months (β = 0.34,95%CI[‐0.35–1.02],*p* = 0.329). There were no associations between LTF and global amyloid burden. However, higher EC tau was associated with accelerated 1‐week LTF (β = ‐0.18,95%CI[‐0.31—0.05],*p* = 0.009) but not with extended LTF(β = ‐0.14,95%CI[‐0.35–0.07],*p* = 0.172). In contrast, higher IT tau was associated with accelerated LTF after 6 months (β = ‐0.38,95%CI[‐0.75—0.01],*p* = 0.045), but not after 1 week (β = ‐0.15,95%CI[‐0.43–0.13],*p* = 0.280) (Figures 1‐2).

**Conclusion:**

We showed that 1‐week LTF is associated with initial learning and EC tau, and LTF at an extended interval of 6 months was associated with IT tau. This suggests that accelerated LTF may be an early cognitive sign in preclinical AD, but that assessing LTF over different time intervals may reveal unique information.